# A Review of Digital Health and Biotelemetry: Modern Approaches towards Personalized Medicine and Remote Health Assessment

**DOI:** 10.3390/jpm12101656

**Published:** 2022-10-05

**Authors:** Ștefan Sebastian Busnatu, Adelina-Gabriela Niculescu, Alexandra Bolocan, Octavian Andronic, Anca Mihaela Pantea Stoian, Alexandru Scafa-Udriște, Ana Maria Alexandra Stănescu, Dan Nicolae Păduraru, Mihnea Ioan Nicolescu, Alexandru Mihai Grumezescu, Viorel Jinga

**Affiliations:** 1 Department of Cardiology, University of Medicine and Pharmacy “Carol Davila”, 050474 Bucharest, Romania; 2 Department of Science and Engineering of Oxide Materials and Nanomaterials, Politehnica University of Bucharest, 011061 Bucharest, Romania; 3 Research Institute of the University of Bucharest—ICUB, University of Bucharest, 050657 Bucharest, Romania; 4 Academy of Romanian Scientists, Ilfov No. 3, 050044 Bucharest, Romania

**Keywords:** digital health, mobile health, telemedicine, medical biotechnology, biotelemetry devices, Internet of Things, remote patient monitoring, artificial intelligence, precision medicine, personalized medicine

## Abstract

With the prevalence of digitalization in all aspects of modern society, health assessment is becoming digital too. Taking advantage of the most recent technological advances and approaching medicine from an interdisciplinary perspective has allowed for important progress in healthcare services. Digital health technologies and biotelemetry devices have been more extensively employed for preventing, detecting, diagnosing, monitoring, and predicting the evolution of various diseases, without requiring wires, invasive procedures, or face-to-face interaction with medical personnel. This paper aims to review the concepts correlated to digital health, classify and describe biotelemetry devices, and present the potential of digitalization for remote health assessment, the transition to personalized medicine, and the streamlining of clinical trials.

## 1. Introduction

The technological revolution has brought new performance levels, enabling the development of smart interconnected devices and innovative applications that are beneficial in many domains. As health monitoring is an attractive area of research, important technological progress has been reported in the medical field for supporting quality patient care [1,2]. Specifically, intelligent devices, such as smartphones and tablets, are already used by doctors and nurses, employing special applications suitable for the clinical setting [3]. Moreover, utilization of such technologies is not restricted to healthcare facilities, as a broad range of medical services and treatments are publicly available over long distances. Nowadays, professional consultation, instrumentation, monitoring, and even application of a medical procedure or test can be performed remotely using wireless biotelemetry devices [4,5]. Furthermore, emerging tools such as artificial intelligence, Big Data, and the Internet of Things can be involved in delivering, managing, and monitoring different services, reshaping the field of digital health [6,7,8,9].

The use of digital health practices instead of or alongside traditional processes holds promise for accelerated disease diagnosis, reduction of errors, improved service quality, better health control through more affordable and accessible procedures, provision of real-time updates, and enabling personalized treatments [2,10]. Given their many benefits for both patients and medical personnel, digital health tools have started to gain increasing attention from researchers worldwide, especially in the turbulent times of the COVID-19 pandemic. In this respect, this review proposes a comprehensive path, beginning with setting the framework of discussion by briefly describing the related concepts (e.g., mobile health, telemedicine, biotelemetry, Internet of Things, big data, artificial intelligence). Further, there are presented the biotelemetry devices found at the core of digital health implementation into practice. Moving on, the present paper focuses on the roles of digital health and biotelemetry in remote health assessment, personalized medicine, and clinical trials, with an additional emphasis on the applications in the pandemic context.

For the realization of this paper, relevant English language articles have been retrieved and analyzed from the Science Direct and Google Scholar databases using various combinations of the following keywords: “digital health”, “mobile health”, “telemedicine”, “biotelemetry”, “wearable devices”, “implantable devices”, “ingestible devices”, “injectable devices”, “internet of things”, “artificial intelligence”, “big data”, “remote patient monitoring”, “precision medicine”, “personalized medicine”, “digital clinical trials”, “pandemic”, and “COVID-19”.

## 2. Defining Concepts

Digital health is an all-encompassing term that may denominate a technology, a user experience, a service, a product, a process, an ecological system of itself, and part of the ecological system of health services [11]. By definition, digital health is a technology that “connects and empowers people and populations to manage health and wellness, augmented by accessible and supportive provider teams working within flexible, integrated, interoperable, and digitally enabled care environments that strategically leverage digital tools, technologies, and services to transform care delivery” [12].

The vast field of digital health can be further personalized into two main forms, namely mobile health (mHealth) and wearable devices [13]. mHealth represents the application of smart mobile devices, their components, and related technologies for continually monitoring health. When associated with internet-connected diagnostic devices, mHealth provides innovative possibilities for diagnosing, tracking, and controlling diseases, encouraging the participation of patients in their own healthcare and integrating with their personal health records [13,14].

Another term commonly encountered in the field is telemedicine. Telemedicine denotes a health-related service based on telecommunication and electronic information technologies, including a broad range of uses, such as patient consultations, remote control, telehealth nursing, and remote physical and psychiatry rehabilitation [15]. Telemedicine practices have been proven effective for remote healthcare, being particularly useful in regions with a shortage or absence of adequate clinical care facilities [16].

One domain that has greatly contributed to the evolution of telemedicine is biotelemetry. Biotelemetry can be defined as the transmission of biological or physiological signals at a distance. In more detail, these signals are collected through appropriate transducers, post-processed, and sent to external monitoring or control devices that can interpret the acquired data and impact the decision-making process. The main advantage of biotelemetry is that it allows obtaining a broad spectrum of environmental, physiological, and behavioral parameters without alterations in measurements caused by restraints on the patient [4,17,18,19].

Important advances in biotelemetry can be envisaged by taking advantage of the developments in other technological areas. In particular, the Internet of Things (IoT) has attracted increasing attention for interconnecting various physical devices to facilitate data collection and exchange [20]. IoT enables information storage and real-time sharing between devices or to the cloud, from a physical network based on specific sensors. Applied to the medical field, IoT is often termed Internet of Health Things (IoHT). By dynamic monitoring of the human being and expanding access to quality health, IoHT has the potential to improve the treatments’ effectiveness, prevent risk situations, and assist in the promotion of good health [21]. By connecting objects used on a daily basis (e.g., watches, glasses, jewelry, clothing, and shoes), IoHT allows for collecting personalized digital health data and making it available to all agents involved in the medical care process [13,21,22]. Another connected term, Internet of Nano Things (IoNT), is used when discussing nano-sensors from different objects (called “nanomachines”) linked via nano-networks. The use of nanomachines and IoNT represents a major advancement toward personalized medical treatments tailored specifically to the molecular and genetic characteristics of the individual [23,24].

With the advances in technology, the amount of collected data has increased tremendously. Since the 2000s, the concept of Big Data has been introduced, denominating a collection of data or a combination of data arrays [6]. Big Data can be distinguished from conventional analyses of data samples, as data are captured in a comprehensive manner relative to the phenomenon under study. Moreover, the goal is not only to analyze data for answering questions but also to generate promising new hypotheses, Big Data being an important tool for accelerating research [25]. Big Data are often utilized in association with artificial intelligence (AI), given that data are usually analyzed with the aid of machine learning (ML) tools instead of conventional statistical methods [25,26].

**Figure 1:**
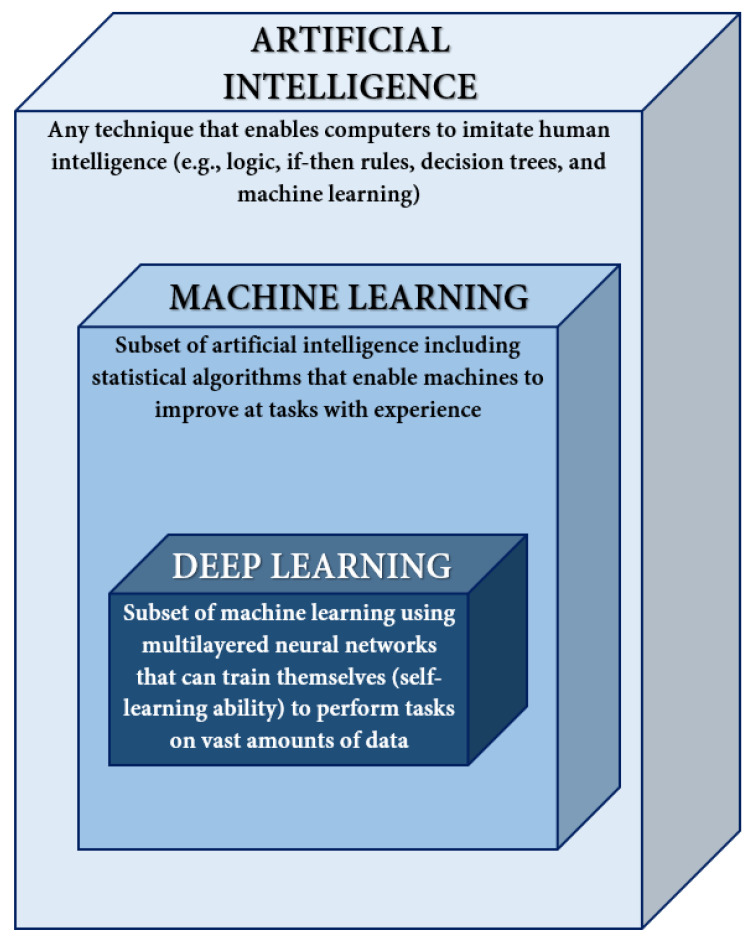
Relationship between artificial intelligence, machine learning, and deep learning. Adapted from an open-access source [33].

AI is regarded as a set of technological solutions (e.g., information and communication infrastructure, software, data processing, decision-making services, and tools) that can imitate intelligent human behavior through the convergence of computer science, statistics, algorithms, information retrieval, and data science. AI provides machines with intelligent problem-solving capabilities, such as planning, reasoning, perception, independent learning, or decision making, when presented with numerous data forms [27,28,29,30]. On the other hand, ML represents a subset of AI that uses statistical techniques that enable computers to improve their predictions and performance concerning a specific task. Moving further, deep learning (DL) is a subdiscipline of ML employing algorithms that can train themselves through a sequential chain of pivotal features from input data [31,32,33]. The relationship between AI, ML, and DL has been schematically represented in Figure 1.

## 3. Biotelemetry Devices

**Figure 2:**
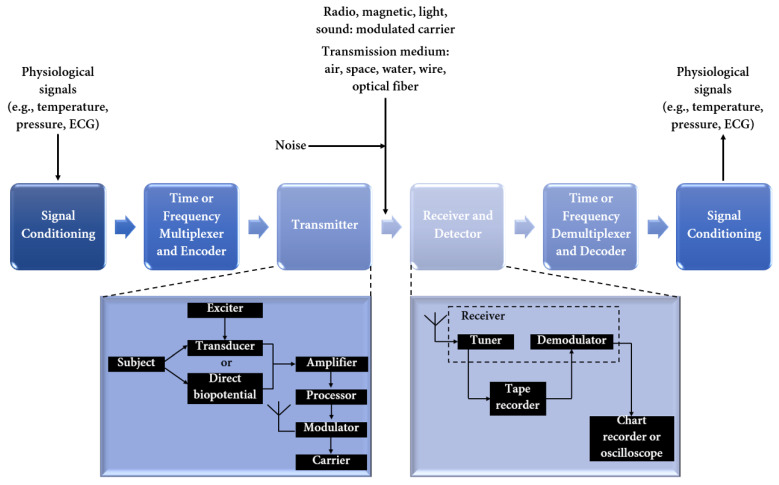
Block diagram of a biotelemetry system. Created based on information from [18,37].

Miniaturized wireless biomedical devices have gained increasing popularity with the proliferation of mHealth. Incorporating many components (Figure 2), bioelectronic devices can sense certain physiological parameters, collect sensitive information, transmit it to a detector, and further affect the human body through stimulation and drug delivery [34,35,36].

Biotelemetry systems can serve in the prediction, detection, diagnosis, and monitoring of various diseases based on the incorporation of wireless medical sensors able to record and transmit a wide range of parameters. Temperature, pressure and strain, optical, chemical, and electrophysiological sensors can capture valuable data from the organism, assisting in the solution of drawbacks (e.g., patient discomfort, restricted mobility, increased anxiety) related to wired sensors commonly utilized in hospitals and emergency rooms [21,37].

**Figure 3:**
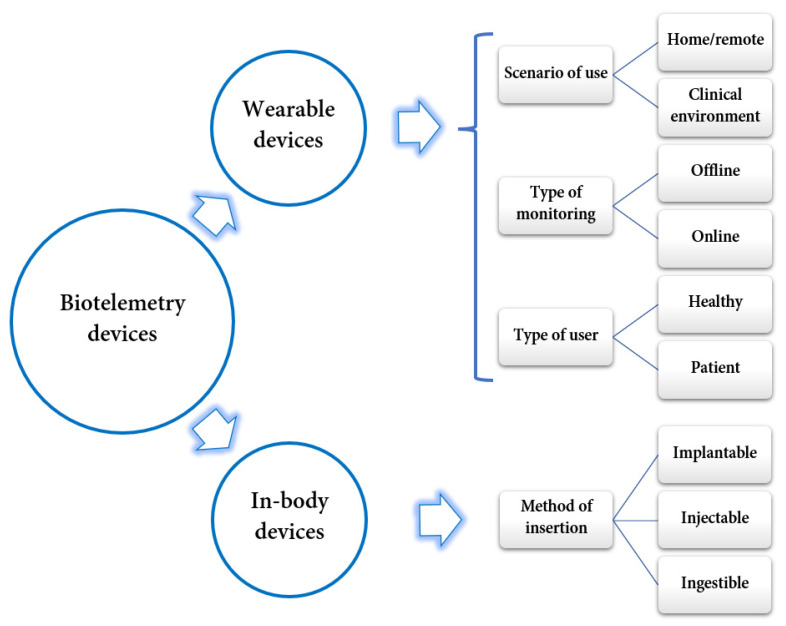
Classification of biotelemetry devices. Created based on information from literature references [36,38].

Biotelemetry devices are highly diverse and versatile. Thus, they can be classified according to several criteria, as depicted in Figure 3. The following subsections describe in more detail the two main categories of biotelemetry devices.

### 3.1. Wearable Devices

In the past few decades, wearable electronics for monitoring body signals have gained increasing ground and significantly contributed to developments in the medical field. Numerous wearable sensing technologies are already commercially available and have reached millions of end users. Devices such as smartwatches, armbands, and trackers have become highly popular in recent years, facilitating the expansion of digital health [39,40]. The main advantage of wearable devices is their external placement, as they can be attached to clothes or garments or be directly worn on the skin. Thus, they represent a comfortable method for monitoring physical and biochemical signals and motions, being noninvasive, compact in size, light, sensitive, and autonomous in operation [23,41,42,43,44].

Nowadays, the smartphone has become an important resource for modern healthcare services. This device incorporates programmable sensors (e.g., ambient light sensor, camera, gyroscope, proximity sensor, microphone, digital compass, touch-sensitive screen, accelerometer, and Global Positioning System—GPS) that can be engaged in collecting useful physiological and behavioral information [45]. Through various mHealth applications, smartphones and smart wearables combine components of detection, communication, and popular consumption, being suitable devices for the ongoing monitoring of biomedical variables during daily routines, with high accuracy and reliability [21,46]. Thus, these widely available, intelligent devices can be easily transformed into portable medical kits [45].

Skin-based wearable devices represent appealing solutions for diagnosing and monitoring different diseases based on qualitative and quantitative analyses of skin secretions. Epidermal wearable devices require direct skin contact, being generally termed electronic skin (e-skin). E-skin consists of flexible electronic components that can record information about the patient’s biomedical variables and send collected data to smartphones or other connected devices. In addition, e-skin adapts to the flexibility of the human body, being able to work even when bent, twisted, or stretched. Moreover, e-skin can receive energy from the electrophysiological processes of the human body, enabling its functioning without batteries. This wearable technology is useful for monitoring and diagnosing arrhythmia problems, the heart activities of premature babies, sleep disorders, and brain activity, among other health issues [46].

Numerous other wearable devices are rendered promising for various applications in the medical/healthcare field [47], including patches [48,49], bandages [50], electronic textiles (e-textiles) [51,52,53], earphones [54,55], contact lenses [56,57,58], ocular rings [59], and more.

### 3.2. In-Body Devices

The miniaturization of technology has led to the emergence of noninvasive microdevices that can not only be worn on the human body but can also be inserted inside it, being able to explore and manipulate complex biological microenvironments [60]. From the point of view of their insertion method, in-body biotelemetry devices are of three main types: implantable—placed into the body during surgery, injectable—injected underneath the tissue, and ingestible—ingested by the patient in the form of a capsule [34].

Implantable devices represent the most conventional category of in-body biotelemetry devices as various medical devices have been surgically introduced over the years, ranging from bulky pacemakers to miniature deep brain implants [36].

Alternatively, injectable devices are placed inside the human body via needles. These microdevices have been recently reported for biomedical applications such as sensing and neuro-stimulation [36].

On a different note, ingestible devices resemble regular capsules and can be swallowed similarly to normal pills. Since the development of the first ingestible device (i.e., wireless endoscope) two decades ago, wireless capsules have been integrated with more advanced features [36]. Thus, ingestible biotelemetry devices have been reported to significantly contribute to the advancement of the diagnosis and treatment of gastrointestinal tract-related conditions, depending on their design, ensuring site-specific drug release, real-time imaging, and/or the sensing of gut biomarkers [60]. Interesting possibilities also arise from the encapsulation of miniature robots into ingestible devices, creating programmable tools that, once released in the stomach, can be remotely directed to the desired location to exert their function (e.g., wound closure, drug delivery, elimination of exogenous particles or substances) [61].

To emphasize the variability of in-body biotelemetry devices, several examples have been gathered in Table 1.

Traditionally, in-body devices are powered via batteries. However, this represents an important disadvantage as batteries increase the device’s size, raise concerns about biocompatibility and patient safety, and must be frequently replaced and/or recharged. To overcome these drawbacks, scientists have directed their efforts toward developing battery-less in-body devices through power harvesting techniques. These technologies are based on energy harvesting from environmental or bodily sources, including electromagnetic energy, tissue motion, heartbeat, body thermal gradients, human motion, and glucose oxidization. Alternatively, battery-less devices can be envisioned by enabling fully passive operation. This can be achieved with the aid of an exterior interrogator placed in close proximity to the in-body device (e.g., it could be part of a hat in the case of brain implants or part of a T-shirt in the case of pacemakers) [36].

## 4. Remote Health Assessment

One of the main advantages of mHealth and telemedicine is dealing with patients located in rural, underserved, or remote areas, where there are few or no conservative healthcare services or existing infrastructure [15,45,89]. Digital health platforms offer the possibility of bringing patients and physicians virtually together, without necessitating any physical contact, relieving congested clinical services, avoiding the risk of acquiring infections with pathogens present in the healthcare facilities, and reducing the expenditure on the patient, who no longer pays for travel costs to the hospital [9,15].

One particularly beneficial field is the application of digital health tools for monitoring patients affected by chronic diseases. This category of patients requires continuous follow-up and periodical assessment of their health status, conventionally translating to regular specialist visits. Fortunately, therapy could be performed remotely for some diseases by sending the data recorded on a biotelemetry device to a specialist [90].

For instance, telemedicine is a convenient approach for cancer patients, especially for palliative care and monitoring the adherence to and side effects of oncolytic oral treatment. In the US, palliative care professionals are also allowed to dispense opioid prescriptions to cancer patients using telemedicine services. Moreover, tele-oncology represents a better alternative to physical hospital visits as it helps to prevent these vulnerable, immunosuppressed patients from contracting infections, such as COVID-19, and developing severe forms [3,89].

Cardiovascular diseases also impose constant monitoring, remote health assessment for these patients being a suitable alternative, especially during epidemics or pandemics. Combining the benefits of phone calls, video calls, records from wearable devices, cardiac implantable electronic device checks, and other digital tools, electrophysiologists can convert most clinical visits to remote monitoring [90]. Telemedicine has found several applications in cardiology, including the early prehospital diagnosis of acute myocardial infarction based on transmitted EKG data, monitoring patients with chronic heart failure, monitoring arrhythmias, and transmitting echo images to a level III center for a second opinion [91].

Another vulnerable population that should and can be protected from avoidable outpatient clinic visits is represented by type 1 and type 2 diabetic patients [90,92]. The development of digital devices for glycemia monitoring, such as glycemic holters and micropumps, has facilitated the self-monitoring of glycemia from the comfort of one’s home. Data can be easily collected and rapidly transmitted to the specialist or the general practitioner for further interpretation and feedback. Based on the received information, the healthcare professional could consider eventual therapy modifications or suggest deeper diagnostic/therapeutic urgent investigations [90].

Careful and regular monitoring and therapy adjustments are also necessary in the case of diabetes complications [92]. For instance, diabetic retinopathy represents a frequently occurring microvascular complication of diabetes mellitus, with the increased risk associated with longer disease duration. Raising the burden of blindness in diabetic populations, this comorbidity must be rigorously screened and monitored [93]. In this respect, telemedicine can be involved in gathering data from fundus cameras and other portable devices able to take retinal photos and send them to specialized referral centers for reading. Comparing telemedicine and the standard fundus oculus exam, good efficacy was reported from the utilization of nonmydriatic cameras in terms of sensitivity and specificity [90].

Digital health has also become a relevant and useful approach in the context of health tourism. In more detail, it allows the accomplishment of pre- and post-operative care processes as anesthesiologists and surgeons can perform teleconsultations for assessing health tourists’ status, even when they reside in their home country. Moreover, healthcare professionals at the health tourism destinations can either directly follow their patients’ healing and recovery process by virtually interacting with them, or they can contact the patient’s local primary care providers via teleconferencing and/or telehealth applications [10].

## 5. Personalized Medicine

Digital health constitutes an essential set of aiding tools for transitioning from traditional healthcare management to real-time personalized monitoring and therapeutic care [61], focusing on the individual instead of the population [94]. Personalized digital health has the potential to harness collected data and tailor healthcare for a particular person, increasing equity in medical care and strengthening health systems worldwide [13,95]. Biotelemetry devices offer the possibility of customized care at all stages of the patient’s pathway, ranging from early diagnosis and personalized interventions to individually tailored medical plans [61].

Moreover, this approach also places some responsibility on the patients, making them an integral part of their therapeutic care. Specifically, digital devices empower patients with more control over their health status, allowing them to make better-informed decisions concerning their own health and contribute with their personal data to shared knowledge about disease evolution [61,94,96,97]. Personalized digital health is also a promising strategy for enhancing the health outcomes of patients with both rare and common diseases, given its potential to objectively assess multiple parameters in real time and improve life quality in terms of physical, mental, and social aspects [95,96]. In this respect, personalized monitoring systems should be raised to the next level of performance, being designed according to the patient’s particular needs and enabling special configurations and adaptations to users’ requirements for better quality of life [1].

The utilization of biotelemetry devices for continuous and real-time data collection also has the capacity to capture the progress of a disease in a personalized way, evidencing short periods of rapid evolution that may otherwise go unnoticed between two consecutive visits to the hospital or would not be detected by traditional clinical methods [96]. Moreover, data reported from such smart digital devices may better reflect certain health status parameters, as they are measured noninvasively in the comfort of the patient’s home, instead of being conditioned by the potential stress and discomfort faced within a healthcare facility [39,98].

Using patient-specific clinical data and electronic healthcare records can also help in creating personalized treatments for patients, such as individually tailored dosage forms of certain medicines. For example, with the aid of 3D printing technologies, customized dosages, drug combinations, shapes, sizes, and drug release profiles can be developed to match the exact needs of a specific patient [61]. Furthermore, based on AI-processed information, customized devices can be constructed to be in tune with the anatomic particularities, physiological conditions, and pathological status of patients. For instance, AI technologies have been reported for designing personalized bioprosthetic heart valves, cardiovascular stents, tissue-engineered vascular grafts, prostheses for tumor resection, and cranial and dental implants [33,37,53,99,100,101,102,103,104,105,106]. Thus, feeding AI algorithms with biometric datasets produced from digital health devices has the potential to generate precision-based, personalized approaches for healthcare delivery [97].

## 6. Digital Clinical Trials

Clinical trials represent essential tools for evaluating the efficacy, effectiveness, and safety of new drugs, medical devices, and clinical interventions for the prevention and treatment of human conditions and diseases [107,108]. Traditional in-person clinical trials are generally highly complex, expensive, time-consuming, and burdensome for both staff and participants [108]. Such studies require the conduction of consent processes, data collection procedures (e.g., physical exams, administration of study drug, sample collection, tests such as imaging studies), and numerous visits to a clinical site, thus implying time commitments from the patient, study investigators, and research personnel. In addition, clinical trials also require significant effort from trial sponsors and contract research organizations [109], depending upon the implication and good cooperation of many parties.

The digital revolution faced in many domains can also be an important opportunity and solution for enhancing efficiency and optimizing value in clinical trials. Digital technologies can be leveraged to improve participants’ access, engagement, and trial-related measurements and enable concealed randomized intervention allocation towards reducing associated costs, minimizing complexity, and decreasing the burden [107,108,110].

Digital health approaches have been reported to be useful in clinical trials, being involved in clinical trial networks for mobile data collection and management, protocol design, and other applications [109]. Important amounts of data are being collected during clinical trials, including clinical and demographic data, patient data retrieved from sensors and wearable devices, patient-reported outcomes and images collected via internet-connected devices, electronic medical record data from a vendor application programming interface, and biological samples drawn at home or in a laboratory [107]. To process and interpret all this information, AI and ML tools have gained popularity in trial execution and data acquisition, processing, and analysis in a virtual trial setting [110,111]. For instance, AI-based algorithms can ensure a good match between participants and studies, improve digital data extraction and computational phenotyping, and aid researchers in interpreting the trial findings [107].

Harnessing digital tools for data collection within clinical trials also brings the advantage of the faster transmission of information directly to researchers. This aspect is further reflected in the improvement in the detection of sporadic events or those that are situation-specific and unlikely to occur during a study visit. Moreover, identifying and reporting adverse and safety events with accelerated speed considerably influences the timeliness of completion and reporting of clinical trials [107].

## 7. Digital Health Applications in the Pandemic Context

Although the application of digital technologies in medicine is not new, it has been tremendously accelerated and popularized during the COVID-19 pandemic [3,11,13]. The opportunity to use real-time data offered by digital health has been demonstrated to be highly valuable in improving the prevention and control of the rapidly changing nature of epidemics [12], triggering the use of telemedicine in dealing with the SARS epidemic in 2003 and MERS-CoV ten years later [90].

Given the large scale of COVID-19’s infection spread, a tremendous burden was placed on health systems worldwide, impairing their capacity to deliver services to both patients infected with this virus and to those with other health problems that were not necessarily related to COVID-19 [112,113]. To avoid overwhelming healthcare facilities, the use of telemedicine was encouraged as an effective solution for monitoring patients remotely. Moreover, remote monitoring also helps to reduce the unnecessary exposure of healthcare givers and vulnerable patients to the virus [114].

COVID-19 has been correlated with specific physiological changes that can be monitored by IoT-based devices and wearables. Thus, with the aid of wearable sensors, patients can be remotely diagnosed in the early stages of infection, and their symptoms can be monitored during the quarantine period. By monitoring physiological parameters, such as respiratory rate, temperature, heart rate, and blood oxygen saturation, wearable devices can detect and alert users of a potential infection before developing clinical symptoms through an early detection algorithm (EDA). Thus, an EDA enables users to self-isolate, seek care or diagnostic testing, and take other steps to mitigate the transmission of the infection during the critical period of disease onset [115,116,117,118].

Wearable health sensing and monitoring technologies also offer solutions for patient triage. This can be done by tracking the infection, screening, and classifying each patient to determine the priority of need and proper place of treatment based on the severity of their condition. Therefore, based on remotely collected data, healthcare professionals can decide whether patients require hospital admission or whether they can be treated in improvised settings [112]. In mild cases, patients can even report their vitals from home, reducing the risk of transmission and saving critical hospital resources [116]. One particular strategy in accelerating the speed of screening viral spread, separating mild from severe infections, and supervising the disease is to employ AI and DL approaches [115].

**Figure 4:**
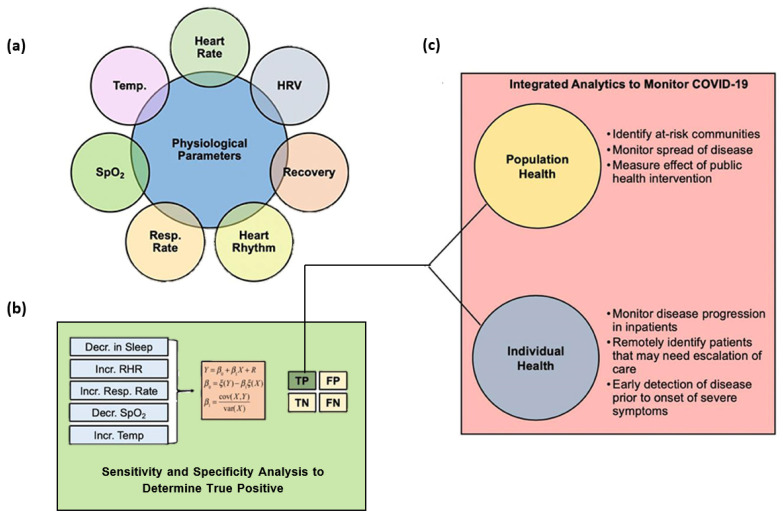
Schematic summary of the roles of wearable sensor technology and predictive analytics for monitoring COVID-19. (**a**) Physiological metrics currently capable of being measured from commercial wearable sensors. (**b**) Changes in physiological metrics that can be inputted into an early detection algorithm for COVID-19 monitoring to ensure that the true positive rate is robust to support the use of the analytics for real-time clinical decision making. (**c**) Uses of integrated analytics in monitoring COVID-19 for individual or population health. Abbreviations: HRV—heart rate variability; Resp Rate—respiration rate; SpO_2_—blood oxygen saturation; Temp—temperature; TP—true positive; FP—false positive; TN—true negative; FN—false negative. Adapted from an open-access source [116].

A visual overview of the roles of wearable biotelemetry devices and predictive analytics in monitoring COVID-19 is offered in Figure 4.

Pre-existing (e.g., TempTraq, Oura ring, Fever Scout, CONTEC, MightySat, PO3M, Fitbit, WHOOP Strap) and new wearable devices have found applications in COVID-19 management, monitoring related physiological parameters such as blood oxygen saturation, heart rate, blood pressure, and body temperature [112,115,116]. In this respect, Table 2 briefly summarizes several recent studies focused on the remote monitoring of COVID-19 patients.

In addition to diagnosing and monitoring disease development in affected people, digital tools were also used to improve contact tracing. Traditionally, this process was performed through interviews, yet this method consumed much time and constituted a source of many human errors. Alternatively, the use of tracking apps, mobile phones, wearables, and some powerful computational methods led to a more accurate depiction of physical interactions between infected people and their contacts, also providing solutions for maintaining social distance [115].

## 8. Discussion

Technological advances have undoubtedly contributed to the recent progress of healthcare services. The miniaturization of low-cost, high-speed communication electronics and the development of user-friendly devices have expanded the application of digital health. Employing wireless sensors, biotelemetry devices improve patient comfort, allowing effortless monitoring and being imperceptible throughout daily activities. Moreover, the opportunity for the rapid and efficient transmission of the retrieved information to remote specialists or general practitioners has enabled faster responses in treating identified health conditions. Communication of health records over long distances has proven to be of great use in the pandemic context, telemedicine practices being an effective solution to reduce the viral spread in the community and protect vulnerable patients from contracting the disease.

By successfully combining records from biotelemetry devices with the computing power of AI tools, promising improvements can be achieved in preventing, diagnosing, and predicting the evolution of diseases based on the continuous monitoring of relevant physiological and behavioral parameters. These advanced methods pave the way for a transition from traditional to personalized medicine, identifying the needs of each patient and tailoring the treatment to their anatomic particularities, physiological conditions, and pathological status.

Despite the recent advances in the interconnected fields of digital health and biotelemetry, there remain some challenges to be solved before utilizing these technologies at their full potential. One of the main problems is represented by the security of data. An increasing number of patients understand the benefits of digital health technologies and agree to contribute to their health records to improve treatments and health system management as long as their privacy is safeguarded [3]. The concern arises from the fact that sensitive patient data and medical records can be shared in the event of a breach in the digital network’s security or can be maliciously used by cyber-criminal groups [6,115]. Thus, before the broad adoption of these technologies, the devices employed must be minimally secure in the face of evolving threats to gain the trust of involved parties [34].

In addition, numerous wearable devices are still in the prototyping phase, requiring further in-depth testing concerning their usability, functions, safety, security, and user acceptance before being accepted on the market [115]. Moreover, the vast diversity of technologies, applications, and terminologies has impeded the creation of a single homogeneous, collaborative system. Thus, there is still a need for an underlying system structure that would facilitate the acceptance of digital tools by doctors, patients, and organizers [45].

One more limiting aspect hindering the large-scale development of digital health comes from the regulatory framework. More specifically, the regulatory development is not able to keep pace with the technological revolution. The regulatory authorities have taken a cautious risk-based strategy regarding the regulation of mobile healthcare applications, exercising their ‘enforcement discretion’ towards the approval of medical devices that pose a minimal risk to patients and consumers. However, digital health represents a rapidly evolving field, necessitating evolution in the regulatory frameworks considering a broad range of medical applications and regulatory harmonization among different regulatory authorities toward creating a homogeneous code of acceptance that would prevent regulations from becoming barriers or disincentives to innovation [14].

Another reason for hospitals’ reluctance to implement emerging technologies and practices resides in the large investments often necessary for establishing performant infrastructure. Nonetheless, the cost of technology decreases rapidly, forcing hospitals to adopt digital communication tools that help not only patients but the hospitals themselves, through digitizing asset tracking, employee management, and planning for better operational efficiency [6].

## 9. Conclusions

To conclude, digital health has the potential to revolutionize medicine through its broad spectrum of technologies, devices, and applications. Biotelemetric methods continue to evolve with each technological advancement, optimizing their size, cost, complexity, power, data security, and performance. However, several commercially available devices can already be reliably used for healthcare applications, enabling remote health assessment and paving the way to personalized medicine. Ongoing research has the potential for accelerating diagnosis, facilitating patient triage, enhancing disease monitoring, and planning customized treatment strategies. Solving the interoperability, regulatory, and technical issues faced in this emerging field holds promise for expanding digital health uses to benefit patients and medical personnel.

## Figures and Tables

**Table 1:** Examples of in-body biotelemetry devices.

Category	Device	Roles	References
Implantable devices	Pacemaker	Induce cardiac contractions when intrinsic cardiac activity is inappropriately slow or absent	[62,63]
	Cardioverter–defibrillator	Detect and stop arrythmias by continuously monitoring the heartbeat and delivering electric shock when needed	[64,65]
	Intracranial pressure monitor	Monitor intracranial pressure following brain interventions	[66,67]
	Cardiovascular pressure monitor	Monitor heart failure patients Manage intravascular volume, inotropic therapy, and pump speed following the implantation of a left ventricular assist device	[68,69]
	Deep brain neurosensor	Monitor deep brain neuropotential	[70,71]
	Retina stimulator	Improve image perception capability	[72]
	Cochlear implant	Restore partial hearing	[73]
	Chronic pain stimulator	Reduce pain on-demand	[74]
	Glucose monitor	Monitor glucose levels in real time	[75]
	Drug infusion system	Enable chronic drug administration	[76]
Injectable devices	Neurostimulator	Provide low-frequency pulses for electrical nerve stimulation	[77]
	Glucose sensor	Track blood glucose levels	[78]
	Bion device for hemicrania treatment	Provide occipital nerve stimulation	[79]
Ingestible devices	Capsule endoscope	Transmit high-quality images of the gastrointestinal tract to an external recorder	[80,81]
	Medication adherence sensor	Measure medication ingestion and adherence patterns in real time and relate pharmaceutical compliance to important physiologic metrics	[82,83,84]
	Gastrointestinal disorder detection system	Provide in situ biomolecular detection based on environmentally resilient biosensor bacteria and miniaturized luminescence readout electronics wirelessly connected to an external device	[85]
	pH sensing system for monitoring gastrointestinal health	Assist in clinical diagnosis of gastrointestinal diseases by detecting the pH of the tract in real time	[86]
	Intraabdominal pressure monitoring system	Measure gastrointestinal intraluminal pressure to monitor and improve pressure-guided relief of intraabdominal pressure	[87]
	Drug delivery capsule	Release drugs at a specific location in the gastrointestinal tract	[88]

**Table 2:** Examples of recent studies on the remote management of COVID-19.

Wearable Device	Roles	Ref.
Smart Telehealth–IoT system	Monitor PPG, ECG, EMG, ACG, and AMG Detect unusual breathing patterns and allow the physician to remotely assess them	[114]
H-watch	Measure SpO_2_, HR, RR, temperature, motion, and audio signals Helps to trace contacts	[119]
Smartwatch	Detect COVID-19 in the pre-symptomatic stage based on HR signals	[120]
Hand band IoT system	Monitor body temperature, indoor temperature, and humidity Send an alert when the body temperature exceeds the allowed threshold temperature	[121]
Headset and mask	Monitor temperature, RR, SpO_2_, and HR	[122]
Mask	Monitor RR	[123]
Oura smart ring	Identify the onset of COVID-19 symptoms	[124]
Oura smart ring	Predict and diagnose COVID-19 in 24 h	[125]

Abbreviations: ACG—acoustic cardiography; AMG—acoustic myography; ECG—electrocardiogram; EMG—electromyogram; HR—heart rate; PPG—photoplethysmography; RR—respiratory rate; SpO_2_—blood oxygen saturation.

## Data Availability

Not applicable.
